# Behavioral, Morphological, and Gene Expression Changes Induced by ^60^Co-γ Ray Irradiation in *Bactrocera tau* (Walker)

**DOI:** 10.3389/fphys.2018.00118

**Published:** 2018-02-20

**Authors:** Jun Cai, Hongxia Yang, Song Shi, Guohua Zhong, Xin Yi

**Affiliations:** ^1^Key Laboratory of Crop Integrated Pest Management in South China, Ministry of Agriculture, South China Agricultural University, Guangzhou, China; ^2^Key Laboratory of Natural Pesticide and Chemical Biology, Ministry of Education, South China Agricultural University, Guangzhou, China; ^3^Guangzhou Entry-Exit Inspection and Quarantine Bureau, Guangzhou, China

**Keywords:** *Bactrocera tau*, sterile insect technique, optimal dose, yolk polypeptides, morphological changes

## Abstract

The sterile insect technique (SIT) may reduce pest populations by allowing sufficient amount of irradiation-induced sterile males to mate with wild females whilst maintaining mating ability comparable to wild males. Although the SIT methods are well understood, the optimal sterilizing dose and processing development stage for application vary among species. To ensure effective pest control programs, effects of irradiation on physiology, behavior, and gene function in the target species should be defined, however, little is known about irradiation effects in *Bactrocera tau*. Here, the effects of irradiation on rates of fecundity, egg hatch, eclosion, mating competitiveness, flight capability, morphology of reproductive organs, and yolk protein (YP) gene expression were studied. The results showed that rates of female fecundity and egg hatch decreased significantly (51 ± 19 to 0.06 ± 0.06 and 98.90 ± 1.01 to 0, respectively) when pupae were treated with >150 Gy irradiation. Flight capability and mating competitiveness were not significantly influenced at doses <250 Gy. Ovaries and fallopian tubes became smaller after irradiation, but there was no change in testes size. Finally, we found that expression of the *YP* gene was up-regulated by irradiation at 30 and 45 days post-emergence, but the mechanisms were unclear. Our study provides information on the determination of the optimal irradiation sterilizing dose in *B. tau*, and the effects of irradiation on physiology, morphology and gene expression that will facilitate an understanding of sub-lethal impacts of the SIT and expand its use to the control of other species.

## Introduction

The *Bactrocera* (Diptera: tephritidae) comprises one of the major pests of tropical fruits (Jamnongluk et al., 2003), and as a result of its euryphagy on many agricultural crops and rapid range expansion, the pumpkin fruit fly [*Bactrocera tau* (Walker)] has become a focus of global quarantine and control programs (Yan et al., 2015). *B. tau* occurs over a wide geographical range and causes significant damage to crop plants that results in reduced quality and yield of vegetable and melon crops (Li et al., 2017). Reductions in fruit quality and yield are caused by larvae, which emerge from eggs laid on the fruit surface and feed inside the fruit (Li et al., 2017). As a technique with proven high specificity and persistence in controlling other species of Diptera pests, the sterile insect technique (SIT) may be a leading method to reduce populations of *B. tau*.

The SIT is species specific, has no negative non-target effects, and successfully eradicated tephritid fruit flies (Pereira et al., 2013). SITs could reduce pest populations by decreasing the hatch rate of eggs through releasing large amounts of irradiation-induced sterile males, which have a comparable competitive mating ability to wild males, to mate with wild females. Insect irradiation is an ideal “genetic” treatment, because the efficacy doses against most insects and mites do not affect the quality of the protected commodities (Follett, 2004). The International Consultative Group was the first group to formally recommend genetic treatment of insect pests, and, based on irradiation data for many tephritid fruit fly species and a limited number of other insect pests, the group proposed a dose of gamma irradiation of 150 Gy for fruit flies and 300 Gy for other insects (Follett, 2004). However, subsequent work has shown that effective doses of irradiation vary among species. For example, 232 Gy is recommended for oriental fruit moth (*Grapholita molesta*) (Hallman, 2004), whereas, a dose of 100 Gy has been shown to provide a high level of quarantine security against *Ceratitis capitata*, by preventing the emergence of adult (Torres-Rivera and Hallman, 2007). A minimum dose of 85 Gy has been suggested for the treatment of *B. tau* in fruit and vegetables (Zhan et al., 2015), where adult eclosion is prevented. An effective sterilizing dose for *B. tau* has not yet been defined. It is important that the mating rates of wild female *B. tau* with irradiated and control males are not skewed, because reductions in the mating fitness of irradiated males compared with wild males would require larger numbers of insects to be released to avoid failure of a control program (Kean et al., 2011).

Irradiation may induce sub-lethal molecular or biochemical impacts that cause a cascade of physiological changes. Variations in gene and protein levels in response to radiation have been found to contribute to radioresistance or radiosensitivity (Suman et al., 2015), for example, heat shock protein is a potential molecular marker due to its high levels of response to irradiation (Shim et al., 2009). It has been suggested that irradiation on pupae at a sterilizing dose could influence expression levels of the responsive genes in the subsequent adult age (Chang et al., 2015). Following X-ray irradiation on *B. dorsalis* pupae, some of the alter proteins act in central energy-generating and in pheromone-signal processing pathways that may contribute to an overall reduction in survival and mating ability (Chang et al., 2015). During the period of vitellogenesis, insect oocytes are packed with yolk proteins that are vital for the development of embryo through the provision of nutrients (Hansen et al., 2014). It is likely that variation in expression of yolk protein following irradiation was closely related to the reproductive ability in insects. Therefore, it is important to understand these, currently unknown, effects of irradiation on *B. tau* behavior, physiology and morphology prior to undertaking a SIT program.

Our study showed that the dose of 150 Gy (or higher) irradiation on the final stage of female pupae could lead to significant decrease in fecundity and hatch rate. When such treatment was applied to male pupae, the hatch rate also decreased after they had mated with untreated females, achieving sterilization. As long as the applied dose was under 250 Gy, there were no effects on the ability of flight capability and mating competitiveness. When irradiation was conducted on pupae, the reproductive organs of female adult flies appeared to be shrink, compared with control, while the morphology of male flies did not show any obvious change. This study could provide information for the validation of sterilizing doses and processing stages in *B. tau* using irradiation. Potential sub-lethal impacts following irradiation treatment were also recorded that could provide a feasible technology package for control of *B. tau*.

## Materials and methods

### Insect rearing

Pumpkin fruit flies were provided by the Department of Entomology at South China Agricultural University and kept in insect cages (30 × 30 × 35 cm) at 25°C and 75% relative humidity, with a 12 h: 12 h (light:dark) photoperiod. An artificial diet of sugar:yeast powder (1:1) and water was provided, and the diet was renewed every 3 days. When the flies reached sexual maturity at 25–30 days post-emergence, they were fed pumpkin and the adult rearing process followed that described (Shen et al., 2014).

### ^60^Coγ ray irradiation treatments

The irradiation treatments were conducted at Guangzhou Furui High-Energy Technology Co., Ltd. When the irradiation was applied to adult flies, the newly emerged flies (<24 h after emergence) were examined, and divided into groups comprising 200 flies that were irradiated with ^60^Coγ at 400 Gy. Three independent groups of flies were irradiated. The female and male flies were irradiated separately. When the treatment was conducted on pupae, three replicates of three groups of 200 pupae were irradiated with 150, 200, or 250 Gy at 2 days before emergence, respectively. Following irradiation, the flies continued to be reared under normal conditions until they reached sexual maturity, when three replicate group of 50 irradiated flies of both sexes from each irradiated group were chosen at random to mate with 50 untreated flies (50 flies from every independent irradiated group were selected for one testing group). Untreated adults (50 males + 50 female flies) were the control. For recording the amount of laid eggs, an artificial instrument was used to collect eggs was placed from 9 a.m. to 6 p.m. As the peak period for copulating could last for 15 days (25–40 days post-emergence), the egg collecting process was repeated every 3 days until the copulating peak ends, to improve accuracy. Fecundity rate and hatch rates were calculated as described previously (Aye et al., 2008).

We assessed flying capability and eclosion rate of 100 irradiated flies as measures of physiological damage. Following irradiation on pupae at different doses, pupae were placed in plastic cylinders (15 cm in height, 6 cm with an upper diameter of 8.5 cm) that were coated with talcum powder on the inside. After the flies were emerged to adult, the rates of eclosion and flight from the cylinder (flight capability) were calculated for females and males separately. Each group was replicated three times to improve accuracy and the untreated flies were set as control group.

We used a high dose (200 Gy) of irradiation to investigate its effects on mating competitiveness and reproductive organs, which represented the highest irradiation level that induced a reduction in fertility and hatch rate, but maintained similar flying capability as wild flies (see section Physiological Changes after Irradiation). For assessing mating competitiveness, after irradiation on pupae, we divided flies into three groups of 75 flies (25 untreated male flies + 25 untreated female flies + 25 irradiated male flies or 25 untreated female flies + 25 untreated male flies + 25 irradiated female flies). For better observation, the treated flies were marked with solution of 0.25% basic fuchsin and 70% ethanol solution prior mating. Mating activity was recorded between 9 p.m. and 11 p.m., and mated pairs were immediately removed. This experiment was performed three times.

### Morphological observation of reproductive organs

For assessing effects of irradiation on reproductive organs, we irradiated 50 pupae with 200 Gy and maintained them until they reached sexually maturity. Once mature, at least 5 female and male flies were selected for dissection to observe the reproductive organs using microscopy (OPTPro2008 Digital microscope imaging system) following methods described by Yi et al. (2014a). Flies were prepared for assessment using SEM by fixing in 2.5% glutaraldehyde mixed with phosphate buffer solution (pH 7.4) followed by incubation at 4°C for 24 h. Flies were then placed in 1% osmium tetroxide for 2 h, washed three times in double-distilled water and then dehydrated in a critical point dryer for 15 min at each graded concentration of alcohol (30, 50, 70, 80, and 90%). The flies were coated with gold prior to assessment by adhering to carbon double sided tape. Reproductive organs were assessed using an FEI-XL30 SEM operated at 20 kV.

### Cloning of *B. tauYP*

The full sequence of the yolk protein (*YP)* gene was cloned using the total RNA extracting from *B. tau*, as described by Yi et al. (2014b). The first strand cDNA was transcribed using the isolated RNA, following the manufacturer's instructions (TaKaRa, China), and the final product was stored at −20°C prior to analysis. The full sequence of the *YP* gene was derived using homologous cloning, where the partial sequence was amplified using the degenerate primers yp-F1, yp-F2, and yp-R1, ypR2 (Table 1). Rapid-amplification of cDNA ends (RACE) was used to obtain the full length of the *YP* gene by following the instructions of GeneRacer Kit (Clontech, US), using the primers listed in Table 1. The amplification process was carried out according to the manufacturer's protocol and sequence verification was carried out by cloning the sequence into a pMD20-T vector (TaKaRa, China) and sequenced completely in both directions.

**Table 1:** Primers used for sequence amplification.

**Section**	**Product size (bp)**	**Name**	**The nucleotide sequence (5′-3′)**
Middle	285	yp-F1	TGCHMAWGTTGCYGGTGCYGC
		yp-R1	ATRCCCATRTADRYRCGYTTGCC
		yp-F2	CGY RTCACHGBYYTGGATCC
		yp-R2	GA YTGGCNKSVACRGCRGGGAA
5′ end	899	yp-5′-1	CACGACCAATCCAGATAACATAC
		yp-5′-2	CCCTTTTCCTTAGCGAACATCTTA
3′ end	508	yp-3′-1	ACTTCTACGTCAACGGTCCAGC
		yp-3′-2	CCCTGGTGCTACCAATGTAATT
		yp-q-F	ATCTGGATTGGCTCGTGGTG
qRT-PCR		yp-q-R	TTCATATTGGTCTATTGAGGTGGC
		actin-F	TTCATATTGGTCTATTGAGGTGGC
		actin-R	CGTGCGTGACATTAAGGAGA

*Product size refers to the number after removing primers and ambiguous end positions*.

### Quantitative real-time (qRT)PCR

The gene expression levels of *B. tau YP* at different developmental stages were investigated following the irradiation treatment on pupae. The samples of RNA were isolated from flies at different developmental stages, including pupae and adults at 7, 15, 30, and 45 days post-emergence. Each treatment had three biological replicates and was transcribed to first-strand cDNA. SYBR green dye (TaRaKa, China) and the primers listed below were used in the qRT-PCR to examine the levels of gene expression. To improve the accuracy, for each biological sample, we performed three times of amplifications to create three technical replicates. For analysis, the method described as Livak was used (Livak and Schmittgen, 2001).

### Data analysis

We tested for differences using one-way and two-way analysis of variance (ANOVA) and *t*-tests. All statistical analyses were computed in SPSS 17.0 software (IBM Corporation, Somers, NY). Data are presented as means ± S.E.Ms.

## Results

### Physiological changes after irradiation

There were no effects of irradiation on fecundity rate when 400 Gy was applied to male flies mated with untreated female flies, however fecundity was lower for irradiated females mated with untreated males compared with control. There were no differences in egg hatch rate between either of the two kinds of treatments and the control (Table 2).

**Table 2:** Effects of 400 Gy irradiation of adults on fecundity and egg hatch rates.

**Treatment group**	**Fecundity (eggs female^−1^)**	**Hatching rate (%)**
CK♂ + CK♀	176 ± 59^a^	95.26 ± 3.18^a^
IR♂ + CK♀	107 ± 39^a^	84.23 ± 5.48^a^
CK♂ + IR♀	26 ± 17^b^	95.04 ± 2.00^a^

*Data are means ± S.E.M. Different letters indicate differences compared to control group (t-test, p < 0.05)*.

There were no effects of irradiation dose on the number of eggs oviposited by untreated females that were mated with irradiated males compared with the control group, when irradiation was applied at the pupal stage. However, there were dose effects for irradiated females that were mated with untreated males, where there was a significant decrease in the amount of eggs laid by treated female flies (Table 3). The results indicated that fecundity decreased significantly only when female flies had been irradiated. There were effects of irradiation at all three doses (150, 200, and 250 Gy) on hatch rates from the two treatment groups (irradiated male + untreated female and irradiated female + untreated male), where fewer eggs hatched from both of the treated groups compared with control (Table 3). The results indicated that irradiation of >150 Gy at the pupal stage could induce significant decreases in hatch rate in the subsequent stage of development.

**Table 3:** Response of adult fecundity and egg hatch rates to different doses of irradiation applied to pupae.

**^60^Co-γ dose (Gy)**	**Treated male** + **Untreated female**		**Treated female** + **Untreated male**	
	**Fecundity (/♀)**	**Hatching rate (%)**	**Fecundity (eggs female^−1^)**	**Hatching rate (%)**
0	51 ± 19^a^	98.90 ± 1.01^a^	51 ± 19^a^	98.90 ± 1.01^a^
150	54 ± 24^a^	0.42 ± 0.21^b^	0.06 ± 0.06^b^	-^b^
200	78 ± 32^a^	0.12 ± 0.01^b^	0^b^	-^b^
250	53 ± 33^a^	0^b^	0^b^	-^b^

*Data are means ± SEMs. Different letters within a column indicate differences at P < 0.05*.

**Figure 1:**
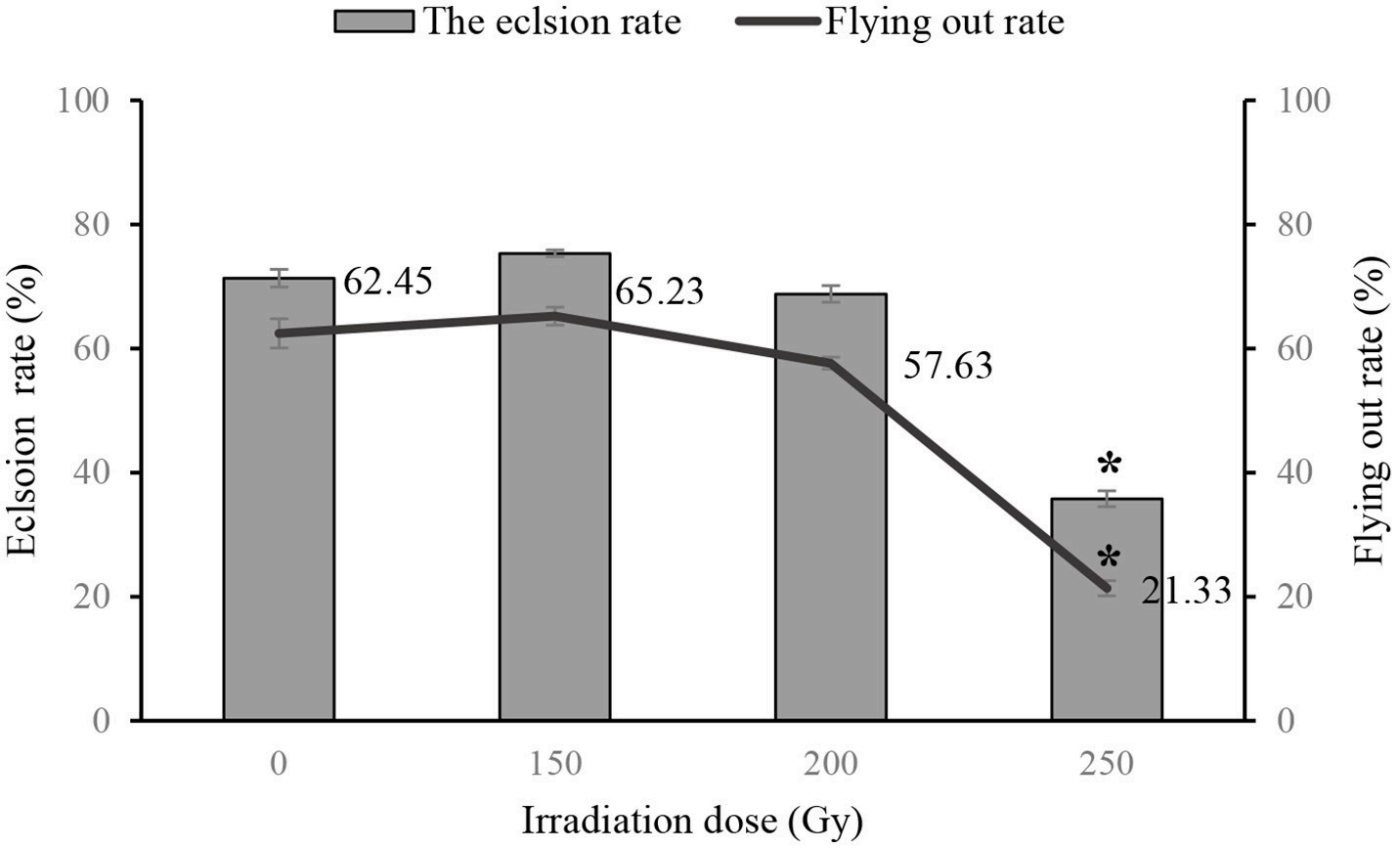
Eclosion rates and flight capablity of flies treated with different doses of irradiation as pupae. ^*^ indicates difference at *P* < 0.05.

There were no differences in eclosion and flying capability of adult flies that had been irradiated as pupae with doses <200 Gy compared with control group, however, both rates (eclosion rate and flying capability) were lower in flies that were irradiated with 250 Gy as pupae (Figure 1). Mating competitiveness of males and females was not associated with irradiation at 200 Gy (χ^2^ = 0.027, *P* = 0.869 and χ^2^ = 2.199, *P* = 0.138, respectively, Table 4).

**Table 4:** Mating competitiveness after irradiation treated on pupae.

**Males**	**Mated (pairs)**	**Not mated (pairs)**	**Females**	**Mated (pairs)**	**Not mated (pairs)**
Treated♂	31	44	Treated♀	28	47
CK♂^a^	31.8	43.2	CK♀^a^	36.9	38.1
Chi-square test	χ^2^ = 0.027, *P* = 0.869 > 0.05		Chi-square test	χ^2^ = 2.199, *P* = 0.138 > 0.05	

*CK♂^a^/CK♀^a^ represented the corrected value of the marked mating adults in treated group*.

### Morphological changes of reproductive organs after irradiation

**Figure 2:**
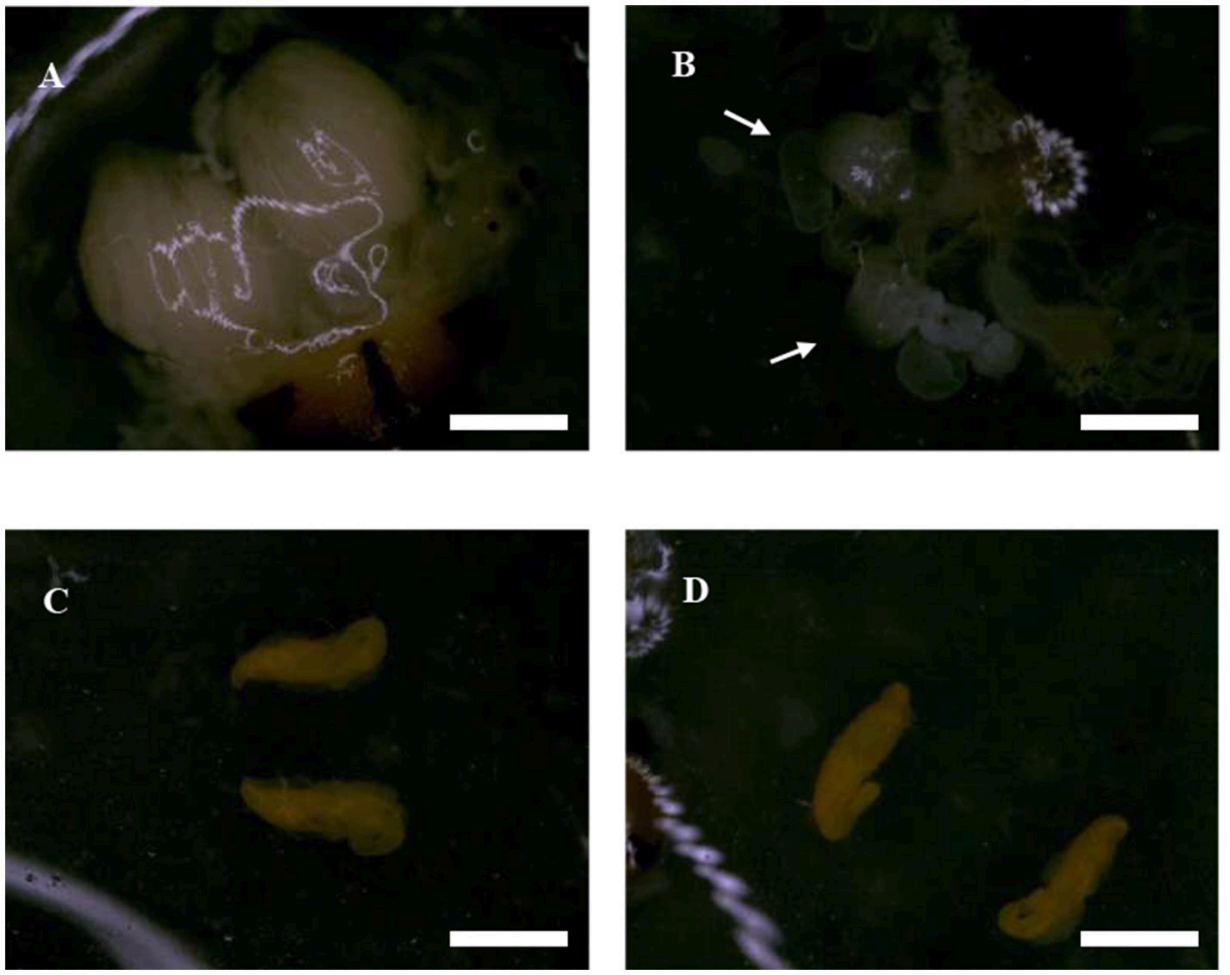
Morphological characteristics of reproductive organs in irradiated and untreated flies observed by optical microscopy. **(A)** Ovary of an untreated female. **(B)** Ovary of an irradiated female. The white arrow indicated the location of ovary. **(C)** Testis of untreated male. **(D)** Testis of irradiated male. Scale bars = 3 cm.

**Figure 3:**
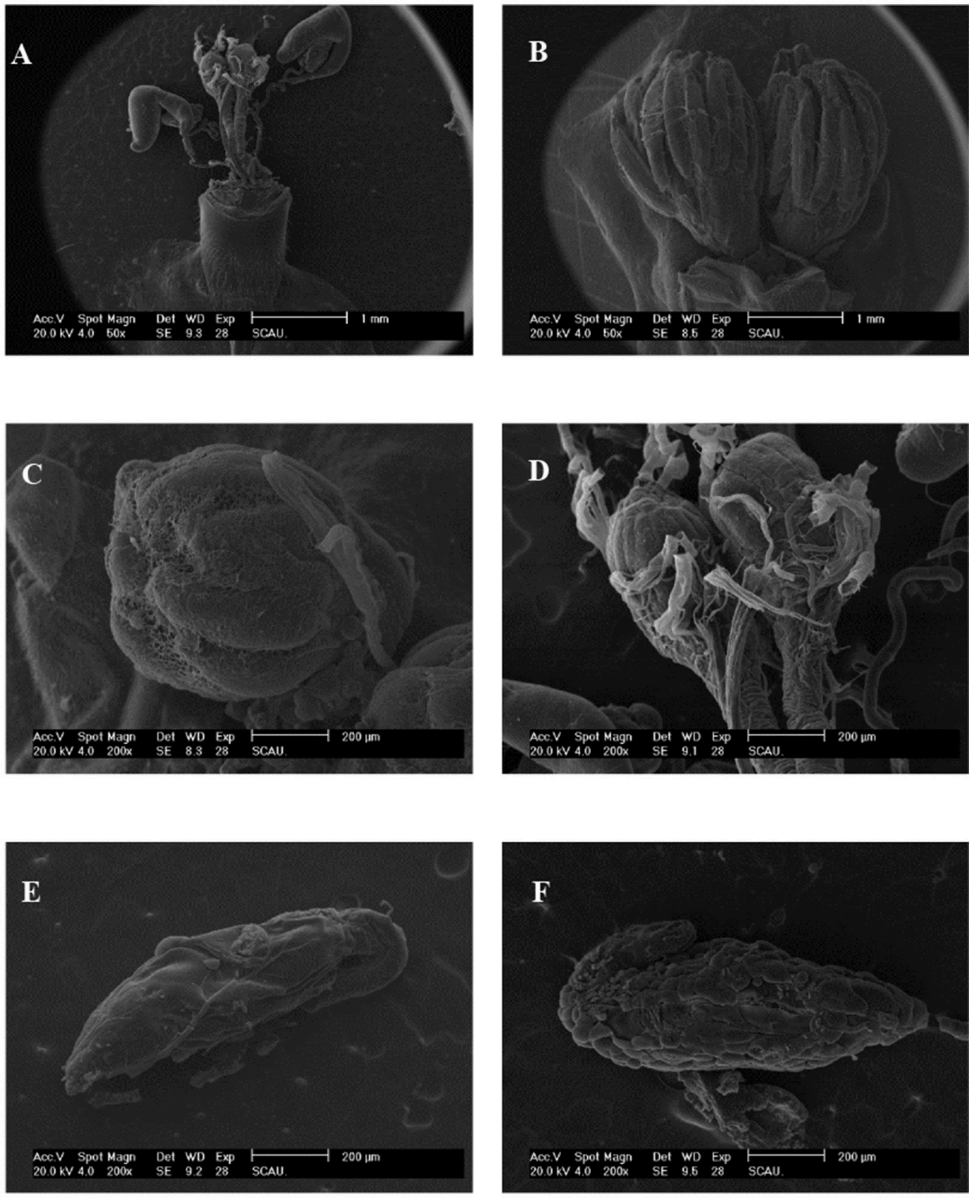
SEM of morphological characteristics of reproductive organs in control and irradiated *B. tau*. **(A)** Complete and **(B)** Intact construction of ovaries of *B. tau* (×50). **(C)** Ovary of an untreated female (×200). **(D)** Ovary of an irradiated female (×200). **(E)** Testis of an untreated male (×200). **(F)** Testis of an irradiated male (×200).

We found that ovaries in the untreated (control) females were well-stocked with eggs that were covered by a transparent membrane, however following irradiation as pupae at 200 Gy, the sizes of ovaries and fallopian tubes were smaller than those of untreated flies (Figures 2A,B). The testes tended to be hippocampus-shaped, yellow in color, and covered with a transparent membrane (Figures 2C,D). SEM imaging of the ovaries (Figures 3A,B) showed they were round in shape and covered with reticular membrane. In the control group, ovaries were dense and well arranged, and separated by ovarioles (Figure 3C), however following irradiation, the shape became flattened, separation by ovarioles was indistinct, and the reticular membrane partially disappeared (Figure 3D). There was no clear difference in shape of testes between the control and irradiated flies (Figures 3E,F).

### Sequencing and expression of *B. tauYP*

**Figure 4:**
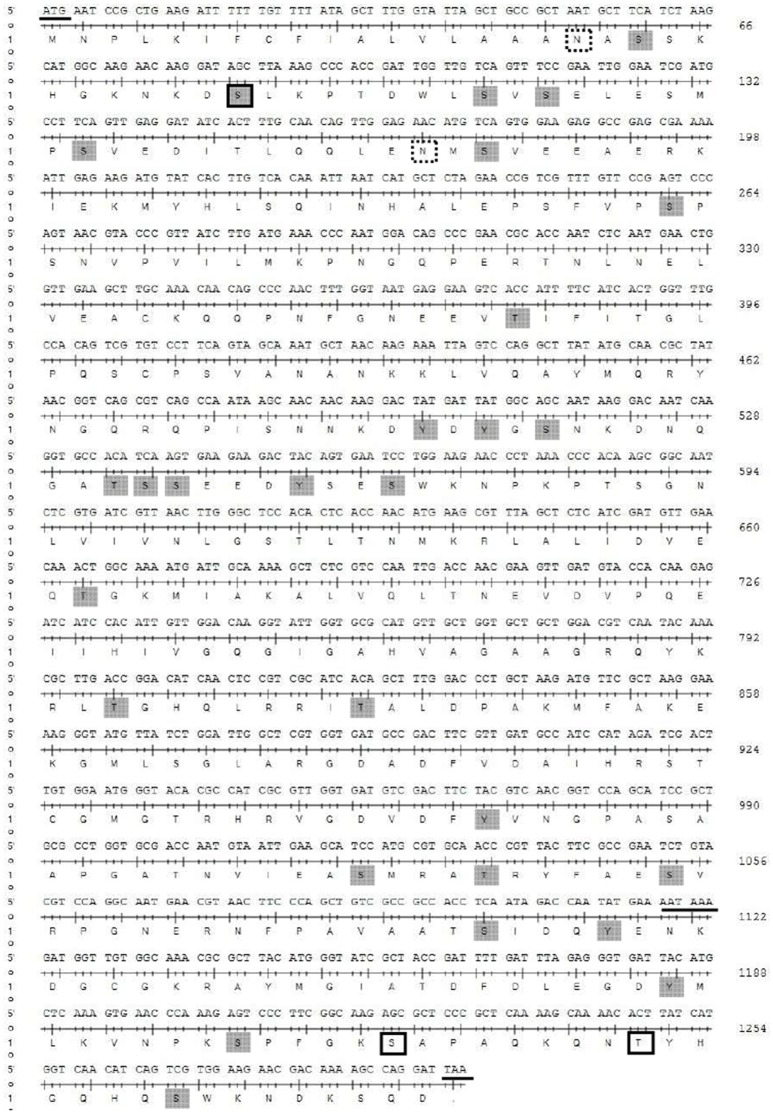
Full length sequence of the *Btau YP* gene. Underline: initiation codon, tailing signal, and termination codon, framed: glycosylation site, and dashed area: predicted phosphorylation sites.

The full-length cDNA of the *YP* gene consisted of 1,296 nucleotides (GenBank accession no. KC985244.1), with an open reading frame encoding 432 amino acids (Figure 4), and exhibited high similarity to the *YP* gene of *B. dorsalis* (88%) and moderate similarity to *A. suspensa* (75%), *Lucilia cuprina* (60%), *Neobellieria bullata* (61%), *D. melanogaster* (56%), and *Musca domestica* (51%), as shown in NCBI BLAST (Figure S1). Phylogenetic analysis showed that the *B. tau* YP had high homology with *B. dorsalis* and *A. suspense* in the Diptera tephritidae. The scale showed the estimates of occurring amino acid site mutations in single locus of each branch (Figure S2).

**Figure 5:**
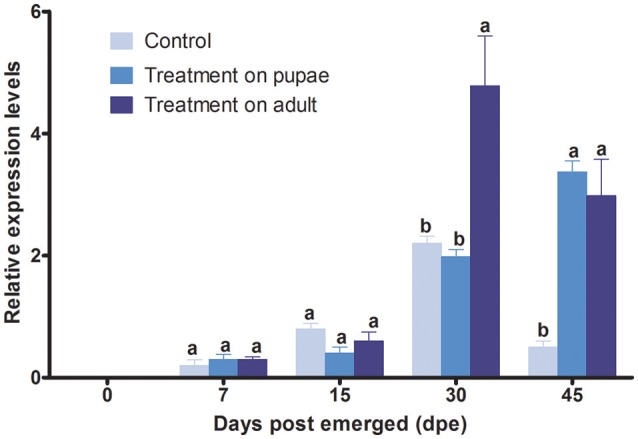
Relative level of expression of *B.tauYP* gene after irradiation, determined by qRT-PCR. The mRAN level of β-Actin transcript was used to normalize the expression level of candidate gene. We tested for differences in mRNA expression level by using two-way analysis of variance (ANOVA). Each point represents the mean value ± S.E.M of three independent. Different letters indicate significant differences in the level of expression at *p* < 0.05. Three biological replicates were performed.

The irradiation treatment applied to the stage of pupae could increase significantly the expression level of the *YP* gene at the 45 days post-emergence (Figure 5). Prior to this time, the level of the *YP* gene expression in treated flies showed no significant change compared with control. When the irradiation treatment was applied to newly emerged adults, the *YP* gene expression level only showed a significant increase at the 30 and 45 days post-emergence compared with control (Figure 5).

## Discussion

Irradiation dose and processing stage of SIT programs are essential for determining the sterile effect (Ramírez-Santos et al., 2017). Optimizing the irradiation dose for inducing sterility is the most important factor for ensuring an effective control program, because insects may not be adequately sterile at low doses and yet less competitive at high doses (Parker and Mehta, 2007). Irradiation at 250 Gy for control of all arthropods in mango and papaya has been recommended and approved for commercial trade (Hallman and Loaharanu, 2002), and a dose of 250 Gy is supported by studies of 34 species of Lepidopteran from 11 families (Hallman et al., 2013). There appear to be variations in response to irradiation doses among *Bactrocera* species. For example, Collins et al. suggested that 70–75 Gy could be used as the lowest practical dose rate for *B. tryoni* (Collins et al., 2008), while 125 Gy irradiation applied to late third instars of *B. dorsalis* could result in no survival to adult stage (Follett and Armstrong, 2004), and a dose of 85 Gy could be applied to late third instars of *B. tau* to prevent adult eclosion (Zhan et al., 2015). Our investigations on the effects of irradiation were initiated to determine whether the treatment doses could cause sterilization and minimize any adverse effects on quality. Irradiated males competition with wild males for females was not reduced. In our study, we showed that male flies irradiated with 150 Gy as pupae were sterilized (the eggs did not hatch after irradiated male mated with untreated female) and their flight capability and mating competitiveness were not significantly affected. We found that irradiation at 400 Gy of newly-emerged adult males did not reduce fecundity or egg hatch rate (Table 2). These results suggest that irradiation of adult males may not lead to complete sterility, which is consistent with a study that showed tolerance to irradiation increased with age and developmental stage (Zhan et al., 2015). Generally, irradiation should be applied at later developmental stages to ensure sterility, such as late stage of pupae, before the somatic cells become fully developed, because the reproductive organs are more sensitive to radiation than other tissues. Another advantage of applying irradiation at the pupae stage is ease of transport. There has been a great deal of research and application of irradiation at the pupae stage due to the convenience of operation and transportation, such as the investigations on *Aedes albopictus* (Oliva et al., 2013), *Melolontha vulgaris* (Oliva et al., 2012), and especially in dipteran insects (Follett and Armstrong, 2004; Zhao et al., 2017). While accounting for factors, such as mating competitiveness, flight capability and the quality of population, this laboratory study indicates that the application of gamma irradiation at doses between 150 and 200 Gy to the pupae stage of *B. tau* could ensure sterilization in a control program.

We observed significant morphological changes in the ovaries following 200 Gy irradiation, but not in the testes. These results are supported by previous reports that also showed irradiation did not consistently cause differences in morphological features (Kheirallah et al., 2017). The reduction in the size and functionality of the female reproductive organs observed in this study would probably lead to reductions in overall fecundity and fertility (Sachdev et al., 2017; Stringer et al., in review). Consistent with the results of male flies in our study, Paoli et al. showed that there were no differences in the ultrastructure of non-irradiated and irradiated *Rhynchophorus ferrugineus Oliv* sperm, except for some abnormalities in maturing spermatids (Paoli et al., 2014). The results from this study suggest that 200 Gy-irradiation may not induce significant morphological damage in male reproductive organs, but cause abnormalities in female reproductive organs that may lead to a decrease in oviposition activities. We were unable to establish the impact on fertilization of irradiation using morphology assessments, so we recommend further work on the effects of irradiation on the reproductive system in this species, particularly, the variations in the number, viability, and motility of sperm.

We hypothesized that irradiation on pupae would elicit sub-lethal effects that would be unobservable at the behavioral level, but would be detectable in mRNA level, which may lead to changes in protein function and abnormal behavior. We found that the *YP* gene from *B. tau* was conserved among *B. dorsalis, A. suspensa* and *N. bullata*. Large amounts of *YP* gene expression following a blood meal reflect large scale protein synthesis to provide essential nutrients required for embryonic development in mosquitoes and reveal the vital role of YP in the development of vitellogenesis (Hansen et al., 2014). In adult female *D. melanogaster*, there is an increase in the synthesis and secretion of yolk polypeptides occurring during the first 24 h following eclosion (Jowett and Postlethwait, 1980). After it is synthesized in fat body, YP protein is secreted into the circulatory system and forms oligomers that are transported into the egg, by endocytosis, to form yolk granules, and then hydrolyzed to amino acids or small peptides by protease (Hung and Wensink, 1983; Raikhel and Dhadialla, 1992). In this study, *YP* gene expression increased significantly at 30 and 45 day post-emergence when irradiation had been applied to pupae, compared with control group (Figure 5). Previous work has shown that 100 Gy of X-ray irradiation also led to a 3.4-fold increase in the expression of profilin, a protein involved in spermatogenesis (Chang et al., 2015). Despite the higher transcription level of *YP* gene compared with control, in this study, there was a decrease in the fecundity that may have been caused by lower amounts of active YP protein or the inhibited effects on oocyte maturation by the irradiation (Rondaldson, 1995). Such hypothesis needs further demonstration by proteinomics study. Moreover, many researches pointed that the poor correlation between mRNA and protein is not unusual, and the transcription and translation process are no where close to linear (Maier et al., 2009; Chang et al., 2015). As many factors during the process could have influences on the efficiencies of translation of proteins, including RNA secondary structure, condon bias and ribosome occupancy (Maier et al., 2009).

In this study, we investigated the optimal sterilizing dose and processing stage of irradiation in *B. tau* by evaluating rates of fecundity, egg hatch, eclosion, mating competitiveness and flight capability. We then assessed the effects of irradiation on morphology of reproductive organs and found that while there were changes in the ovaries of irradiated females, there were no effects on the testes of irradiated males. We found expression of the *YP* gene increased in response to irradiation at specific growth stages, but the mechanisms were unclear. Overall results showed the influence of irradiation on physiology, morphology and gene expression, but additional efforts should be made to finally confirm the effective sterilizing dose for *B. tau*.

## Author contributions

JC and SS performed the experiments; HY analyzed the data, and XY and GZ wrote and revised the manuscript.

### Conflict of interest statement

The authors declare that the research was conducted in the absence of any commercial or financial relationships that could be construed as a potential conflict of interest.

## Supplementary material

Figure S1 Homologous comparison of YP deduced amino acid sequences between *B. tau* and other flies. The sequences were obtained from GenBank. Bd-yp (*B. dorsalis*, AAM00372.1), As-yp (*A. suspense*, AAC01961.1), Lc-yp (*L. cuprina*, ACY56509.1), Nb-yp (*N. bullata*, AAS75325.1), Dm-yp (*D. melanogaster*, AAL68367.1), and Md-yp (*M. domestica*, CAA65731.1).

Figure S2 Phylogenetic tree of *B. tau* YP. Accession numbers are *D. primaeva* AAB06034.1, *D. truncipenna* AAC47253.1, *Calliphora vicina* CAA50066.1, and *Glossina morsitans* AAP84615.1.
