# Supplementary figures and images for: Behavioral, Morphological, and Gene Expression Changes Induced by 60Co-γ Ray Irradiation in Bactrocera tau (Walker)
Source: Front Physiol. 2018 Feb 20;9:118. doi: 10.3389/fphys.2018.00118 (PMC5826196; doi:10.3389/fphys.2018.00118)

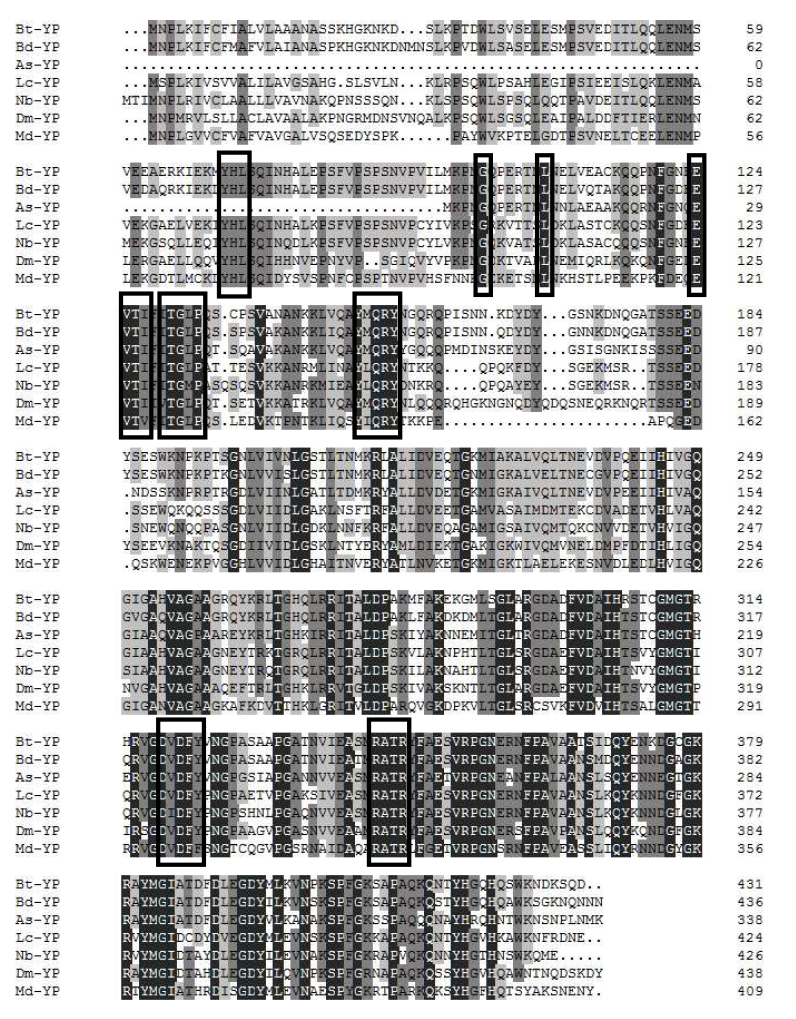

Supplement: Figure S1 — Homologous comparison of YP deduced amino acid sequences between B. tau and other flies. The sequences were obtained from GenBank. Bd-yp (B. dorsalis, AAM00372.1), As-yp (A. suspense, AAC01961.1), Lc-yp (L. cuprina, ACY56509.1), Nb-yp (N. bullata, AAS75325.1), Dm-yp (D. melanogaster, AAL68367.1), and Md-yp (M. domestica, CAA65731.1). [file Image1.TIF]

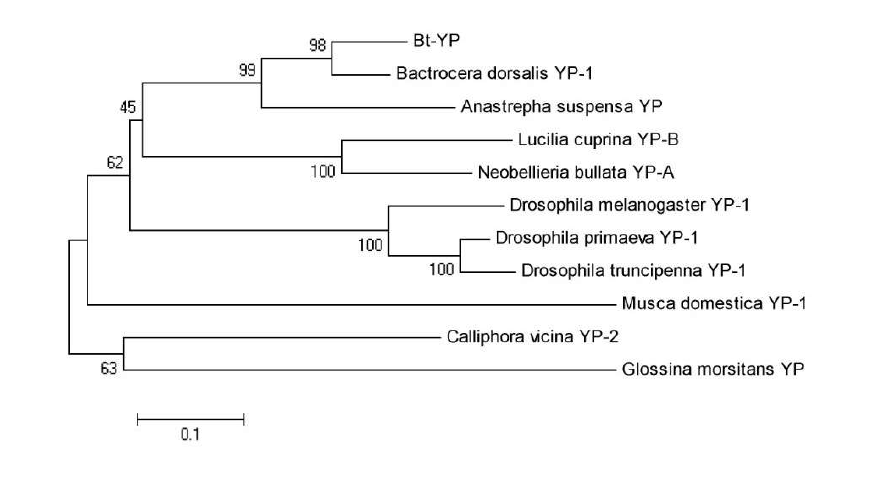

Supplement: Figure S2 — Phylogenetic tree of B. tau YP. Accession numbers are D. primaeva AAB06034.1, D. truncipenna AAC47253.1, Calliphora vicina CAA50066.1, and Glossina morsitans AAP84615.1. [file Image2.TIF]
